# Guiding Treatment With Recovered CTRCD: Time to Focus on Definitions of Recovery and Randomized Data

**DOI:** 10.1016/j.jaccao.2024.09.003

**Published:** 2024-10-15

**Authors:** Brian P. Halliday, Muhummad Sohaib Nazir

**Affiliations:** a National Heart and Lung Institute, Imperial College London, London, United Kingdom; b Royal Brompton and Harefield Hospitals, Guy’s and St. Thomas’ NHS Foundation Trust, London, United Kingdom; c School of Biomedical Engineering and Imaging Sciences, King’s College London, Guy’s and St. Thomas’ Hospital, London, United Kingdom

**Keywords:** chemotherapy, myocardial recovery, precision medicine

**Figure:**
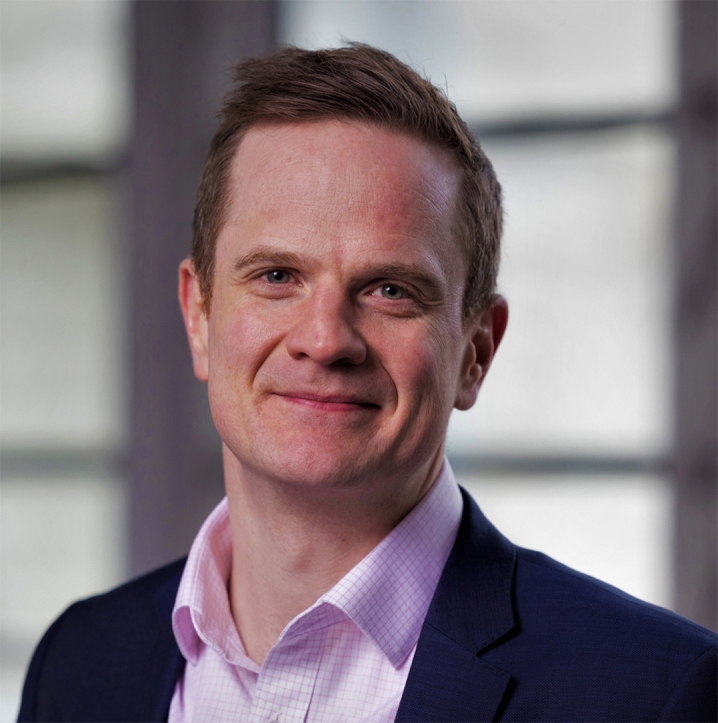

The number of patients receiving therapy for cancer is increasing as life expectancy rises, diagnostic capabilities improve, and therapeutic options grow. As a result, and along with improved surveillance for cardiovascular side effects, the number of people diagnosed with cancer therapy–related cardiac dysfunction (CTRCD) is also increasing.

Many of the current treatment paradigms for CTRCD are taken from the treatment of patients with symptomatic heart failure with reduced ejection fraction, for whom therapy has transformed outcomes over the past 40 years.^1^ However, patients with CTRCD are a unique population comprising various types of cancer therapies, and there are challenges in directly translating evidence from other contexts. The adverse impact of having to interrupt cancer therapy because of CTRCD and the consequent benefit of early treatment to facilitate the continuation of cancer care is an important consideration and requires close multidisciplinary discussions. Stand-alone studies that assess the effectiveness of different therapies and strategies in specific cancer populations are important to guide care and have been the focus of much work in recent years. Several smaller clinical trials have been performed to support the use of cardioprotective agents such as angiotensin receptor blockers and beta-blockers in specific cancer cohorts receiving cancer therapy.^2^^,^^3^ Many important questions remain. Which therapies are most effective at improving outcomes? Does this differ depending on the cause of CTRCD? Which patients gain the most benefit from therapy, and should therapy be permanent in all cases?

The final question is the important topic of the paper by Park et al^4^ in this issue of *JACC: CardioOncology*. They report the outcomes of patients from a single center in Korea who were diagnosed with CTRCD and who subsequently experienced improvement in echocardiography-derived 2-dimensional left ventricular ejection fraction (LVEF). In this retrospective analysis of a prospective, observational cohort, outcomes were reported on the basis of whether all cardioprotective therapies were continued or not after the point of improvement. Cardioprotective therapies included guideline-directed medical therapies that are known to improve prognosis in patients with heart failure with reduced ejection fraction as well as statins.^5^^,^^6^ Those who withdrew or down-titrated any cardioprotective therapies were considered in the withdrawal group. The primary endpoint was a composite of hospitalization for heart failure and a reduction in LVEF ≥10%. Changes in LVEF and left ventricular global longitudinal strain were also considered.

The headline result was that among the subgroup of patients with baseline LVEFs <45%, withdrawal of therapy following improvement in LVEF was associated with a higher incidence of the composite primary endpoint. Within this relatively small subgroup, the final LVEF was about 5% lower in those who withdrew therapy compared with those who continued therapy. Similar findings were not present among those with less severe left ventricular dysfunction at baseline. The investigators should be congratulated for these interesting hypothesis-generating findings. A few points are worth discussing in greater detail.

We should acknowledge the inevitable sources of systematic bias in this retrospective analysis. Decisions about therapy withdrawal will not have been taken at random and will have been influenced by factors for which it is impossible to account. Despite the lack of statistical difference between the baseline characteristics of the groups, there are likely to have been important differences between the groups. Variation in follow-up may also have played a role. If the decision was made to withdraw therapy in the clinic, it is possible, if not likely, that echocardiography was performed more frequently and that fluctuations in LVEF were more likely to have been picked up. The number of scans and the timing of scans in each group are not immediately clear. We are therefore unable to definitively conclude that the difference in outcomes is mediated solely through the effect of therapy withdrawal.

The definition of improvement in LVEF is also important to note. The study included any patient who had at least a 10% increase in LVEF from the point of CTRCD diagnosis. This therefore included patients with LVEFs <40% at the point of improvement. It appears from the investigators’ Figure 1 that the association between treatment withdrawal and reduction in LVEF may have been driven by 4 of 19 patients in whom treatment was withdrawn while their LVEFs were <45%. The decision to withdraw therapy is likely to have been driven by individual circumstances in these cases. However, it is unlikely that clinicians would ordinarily elect to trial withdrawal of disease-modifying therapies in patients with persistently reduced LVEFs unless faced with extenuating circumstances. It is therefore difficult to draw information about the magnitude of risk associated with therapy withdrawal among patients with more convincing myocardial remission from the data presented. Additionally, 3-dimensional echocardiography has better precision for the measurement of LVEF in patients with cancer and is recommended for the evaluation of CRTCD, rather than the 2-dimensional echocardiographic LVEF measurements presented in this study.^7^

**Figure 1:**
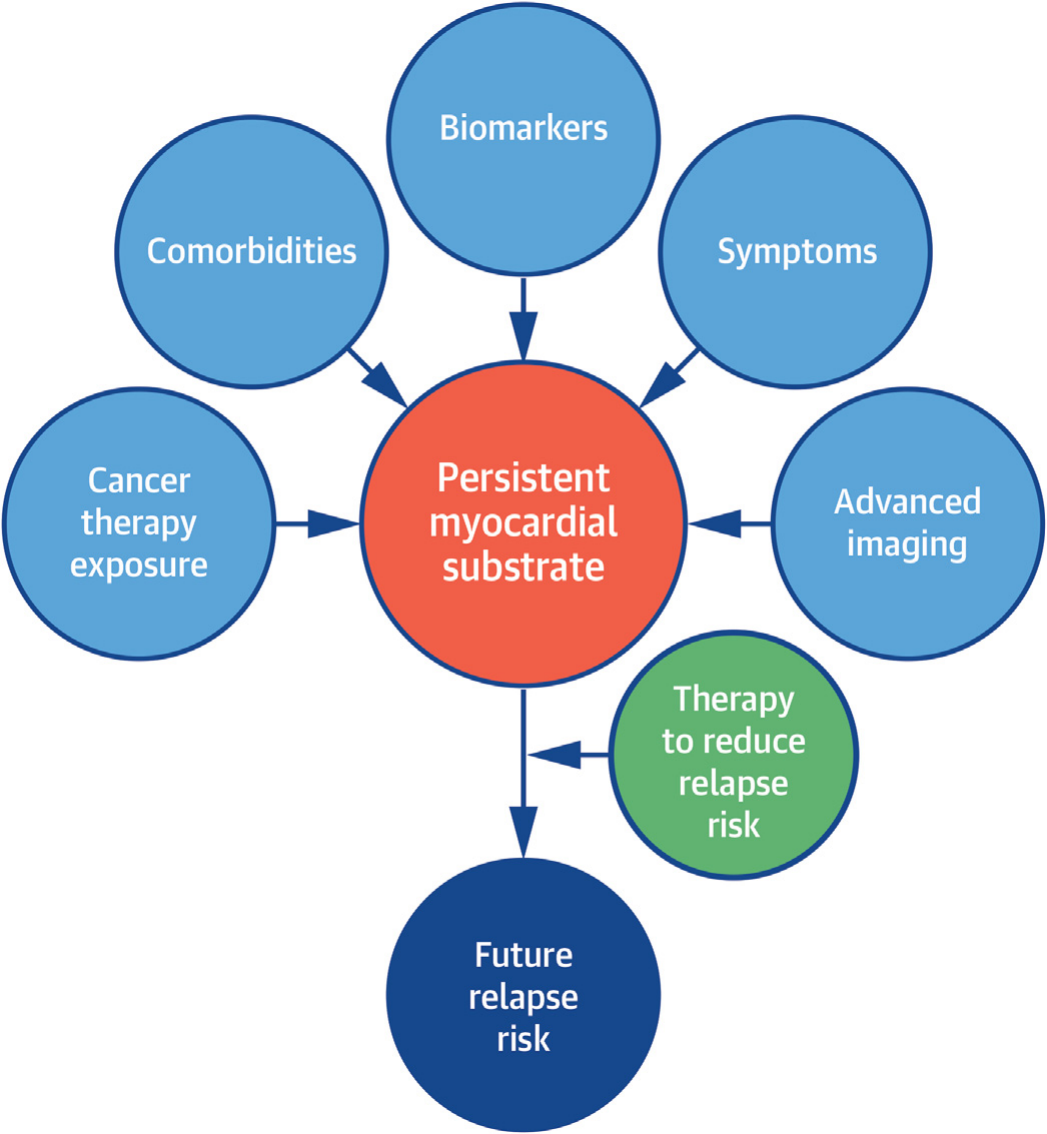
Precision Medicine for CTRCD Recovery This figure illustrates the factors that may determine future treatment decisions in patients following cancer therapy–related cardiac dysfunction recovery. We envisage incorporating clinical, biomarker, genetic, and imaging data to define the precision therapies required to maintain clinical myocardial remission.

It is encouraging to see that there are 3 well-designed randomized trials currently assessing the effect of therapy withdrawal in specific cohorts of patients with cancer relevant for clinical practice (NCT06183437, NCT05880160, ANZCTR12621000928819). The question may be most relevant to those causes of CTRCD considered the most reversible, such as with anti–human epidermal growth factor receptor-2 therapy.^8^ All use robust definitions consistent with true remission of CTRCD. It is important to remember the limitations of defining recovery using a single measure of LVEF^9^ and to incorporate symptoms and biomarkers into a more patient-centered evaluation. Long-term outcomes will be important to collect if therapy withdrawal appears to be safe for the majority in the short term. This is particularly relevant for those who receive cancer therapy early in life. Whether there are robust clinical predictors of future recurrence that can guide the use of therapy will be important to clarify.

It is likely that a proportion of patients have persistent myocardial substrate that increases the risk for future recurrence.^10^ This substrate may be acquired persistent subclinical abnormalities in myocardial function from cancer treatment, coexistent comorbidities, or contributions from intrinsic genetic susceptibility.^11^ Recurrence may occur when patients are exposed to increased cardiac work load, such as during acute infection or pregnancy. Use of a beta-blocker was associated with reduced risk for events in the present study. This observation is consistent with our data from the TRED-HF (Therapy Withdrawal in Recovered Dilated Cardiomyopathy) trial^12^ and is in keeping with the notion that these medications may be most effective at limiting myocardial work load in the context of external challenge. Identifying the medications that are most effective in maintaining cardiac function is an important topic of investigation. Prompt treatment of comorbidities that affect cardiac reserve will also be important to consider. It is notable that diabetes mellitus was associated with increased risk for events in the study by Park et al.^4^

Although not available routinely in the clinical setting, it is possible that the type and extent of persistent myocardial substrate may be characterized by advanced imaging such as with cardiovascular magnetic resonance or positron emission tomography, circulating biomarkers, and genetic testing in the future. This may guide the use of personalized therapies that target specific pathways to prevent future recurrence, following our cancer colleagues in adopting robust, evidence-based precision medicine approaches (Figure 1).

## Funding Support and Author Disclosures

Dr Halliday has received funding from the British Heart Foundation (FS/ICRF/21/26019) and the Rosetrees Trust; and has been an advisor to AstraZeneca. Dr Nazir has reported that he has no relationships relevant to the contents of this paper to disclose.
